# P-1954. Wastewater-Based Surveillance More Accurately Describes Disease Burden Of COVID-19 In Communities with Less Than 60,000 Inhabitants – An Ontario-Wide Study

**DOI:** 10.1093/ofid/ofae631.2113

**Published:** 2025-01-29

**Authors:** Nada Hegazy, K Ken Peng, Joan Hu, Charmaine Dean, Elizabeth Renouf, Robert Delatolla

**Affiliations:** University of Ottawa, Ottawa, Ontario, Canada; Simon Fraser University, Vancouver, British Columbia, Canada; Simon Fraser University, Vancouver, British Columbia, Canada; University of Waterloo, Waterloo, Ontario, Canada; University of Ottawa, Ottawa, Ontario, Canada; Univeristy of Ottawa, Ottawa, Ontario, Canada

## Abstract

**Background:**

The emergence of COVID-19 in Canada prompted extensive efforts in population-wide surveillance, including wastewater-based surveillance (WBS). WBS offers insights into the prevalence of infectious disease burden, however, reasons for poor correlation between hospital admissions and wastewater viral signal in some locations across Ontario remain unclear, while others demonstrate remarkable correlations. This study aims to elucidate the parameters influencing hospitalization-WBS correlation quality to guide infectious disease research and surveillance.

**Figure 1:**
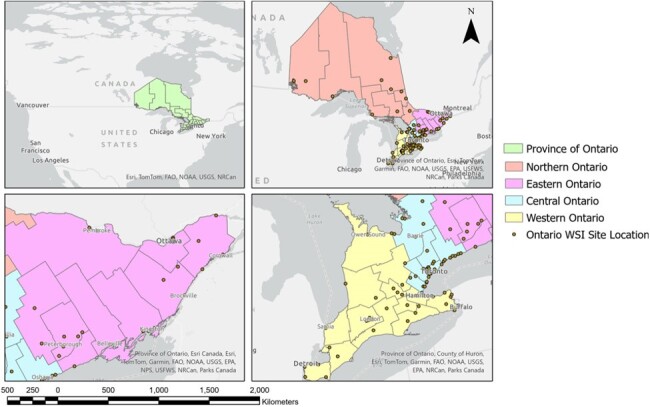
Geographic overview of sampling locations in Ontario, Canada

**Methods:**

Wastewater viral signals and daily hospital admissions geospatially linked with the surveyed sewersheds, were obtained from the Ontario Wastewater Surveillance Initiative. The study included 94 sampling sites (Figure 1), excluding those with inadequate sampling frequency. Spearman’s rank correlation coefficient (ρ) was calculated between hospital admissions and wastewater signals for all included sites during the Omicron BA.1 and BA.2 waves from Nov. 1^st^,2021 to Jun. 30^th^, 2022. Regression trees were constructed using the R packages “rpart” and “rpart.plot” to identify factors influencing hospitalization-WBS ρ, including population size, hospital admissions range, proportion of zero admissions, and site isolation status.

**Figure 2:**
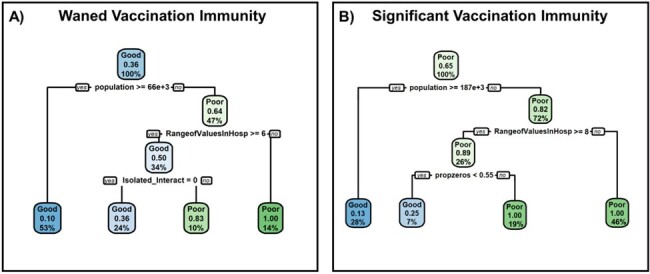
Regression trees representing a classification model for evaluating the correlation quality between hospitalizations and wastewater signal during the Omicron BA.1 wave – coinciding with a period of waned vaccination immunity (A) and the Omicron BA.2 wave – coinciding with a period of significant vaccination immunity (B).

**Results:**

Regression tree analysis identified critical population size thresholds influencing hospitalization-WBS ρ: populations under 66,000 exhibited weaker ρ (ρ > 0.650) during Omicron BA.1, and under 187,000 during the BA.2 wave (Figure 3). Larger population areas exhibited stronger hospitalization-WBS ρ, suggesting both are reliable infectious disease burden indicators, influenced by differences in community immunity dynamics between the two waves.

**Conclusion:**

Populations under 66,000 during a period of waned vaccine immunity and under 187,000 during a period of significant vaccine immunity exhibit distinct relationship between hospital admissions and wastewater signals. These thresholds mark key sizes of communities where the efficacy of wastewater surveillance becomes more pronounced, emphasizing its utility in smaller populations where clinical surveillance of infectious diseases may be limited.

**Disclosures:**

All Authors: No reported disclosures

